# Single-nucleus transcriptomic mapping of blast-induced traumatic brain injury in mice hippocampus

**DOI:** 10.1038/s41597-023-02552-x

**Published:** 2023-09-20

**Authors:** Lingxuan Zhang, Qiuyun Yang, Ruixuan Yuan, Manrui Li, Meili Lv, Lin Zhang, Xiaoqi Xie, Weibo Liang, Xiameng Chen

**Affiliations:** 1 West China School of Basic Medical Sciences and Forensic Medicine, Sichuan University, Chengdu, 610041 China; 2 Department of Forensic Genetics, West China School of Basic Medical Sciences and Forensic Medicine, Sichuan University, Chengdu, 610041 China; 3 West China Second University Hospital, Sichuan University, Chengdu, 610041 China; 4 Department of Immunology, West China School of Basic Medical Sciences and Forensic Medicine, Sichuan University, Chengdu, 610041 China; 5 Department of Critical Care Medicine, Sichuan University, Chengdu, 610041 China; 6 Department of Forensic Pathology and Forensic Clinical Medicine, West China School of Basic Medical Sciences and Forensic Medicine, Sichuan University, Chengdu, 610041 China

**Keywords:** White matter injury, Pathogenesis

## Abstract

As a significant type of traumatic brain injury (TBI), blast-induced traumatic brain injury (bTBI) frequently results in severe neurological and psychological impairments. Due to its unique mechanistic and clinical features, bTBI presents diagnostic and therapeutic challenges compared to other TBI forms. The hippocampus, an important site for secondary injury of bTBI, serves as a key niche for neural regeneration and repair post-injury, and is closely associated with the neurological outcomes of bTBI patients. Nonetheless, the pathophysiological alterations of hippocampus underpinning bTBI remain enigmatic, and a corresponding transcriptomic dataset for research reference is yet to be established. In this investigation, the single-nucleus RNA sequencing (snRNA-seq) technique was employed to sequence individual hippocampal nuclei of mice from bTBI and sham group. Upon stringent quality control, gene expression data from 17,278 nuclei were obtained, with the dataset’s reliability substantiated through various analytical methods. This dataset holds considerable potential for exploring secondary hippocampal injury and neurogenesis mechanisms following bTBI, with important reference value for the identification of specific diagnostic and therapeutic targets for bTBI.

## Background & Summary

Blast-induced traumatic brain injury (bTBI) is a type of traumatic brain injury (TBI) caused by blast shock wave^1^. Militarily, bTBI is the leading cause of casualties and disabilities among active servicemen in conflict area^2^. In daily life, bTBI can also occur as a result of explosions caused by chemical or industrial accidents. Increasing amounts of data show that bTBI is becoming more common as a type of TBI^3–6^. Compared to other types of TBI, bTBI has unique mechanisms of onset, pathological changes, and clinical manifestations. bTBI is primarily caused by the rapid release of shock waves generated by an explosion^7^. In contrast, traditional TBIs result from direct physical impact or deceleration forces without the explosive component. Increased activation and proliferation of astrocytes, as well as periventricular axonal damage detected through immunohistochemistry for amyloid precursor protein, are prominent neuro-pathological findings in bTBI^8,9^. Studies on the mechanism of injury have shown that the neural impact of bTBI is characterized by diffuse, widespread, and spatially variable patterns, which differ from other forms of brain injury^10^. In that case, the prognosis of bTBI is notably more intricate compared to other types of TBIs, including cognitive impairments, memory loss, headaches, and sleep disturbances^11^. Currently, it is challenging to study the diagnosis and treatment of bTBI, and there is no specific target for diagnosis or treatment.

Understanding the mechanisms of secondary injury and neural repair after bTBI is critical to addressing the aforementioned issues. However, the mechanisms are not entirely clear at present. Previous studies have demonstrated that neural stem cells (NSCs) in neurogenic regions play a key role in neural repair after brain injury^12^. Meanwhile, the secondary injury of the hippocampus has been shown to be closely associated with significant aftermath problems, including cognitive impairment, epilepsy, post-traumatic stress disorder, and affective disorder, after bTBI^13^. Consequently, the hippocampus has assumed a pivotal role in bTBI research, yielding significant advancements encompassing injury mechanisms, bTBI-related consequences and diagnosis. For instance, in the realm of secondary injury mechanisms following bTBI, Ratliff *et al*., observed a reduction in dendritic structure complexity among hippocampal neurons in mice^14^, elucidating potential underpinnings for cognitive and memory impairments following bTBI. However, a comprehensive comprehension of the molecular mechanisms underpinning this occurrence necessitates further inquiry. In the arena of post-sequelae research after bTBI, Chen *et al*. identified heightened tau protein phosphorylation triggered by bTBI, mirroring pathological features observed in the brains of individuals with Alzheimer’s disease (AD)^15^. Nevertheless, the intricate molecular pathways governing tau protein signaling subsequent to bTBI remain to be further investigated, as unraveling this intricacy stands to significantly inform future AD preventive strategies. Furthermore, propelled by the relentless progress of neuroimaging techniques, novel methods for brain imaging have surfaced in the domain of bTBI diagnostic research. Davenport *et al*.‘s investigation leveraged diffusion tensor imaging (DTI) to discern neuropathological shifts within the brain post bTBI, serving diagnostic aims^10^. However, this approach still lacks distinctly specific markers tailored for bTBI-induced injuries. In summary, the pursuit of resolutions to the aforementioned quandaries demands a deeper delve into the intricate mechanisms governing hippocampal neuron cell detriment, nuanced fluctuations in bTBI responses, and the quest for injury-specific biological markers.

To address the aforementioned issues, we used a breakthrough research technology in recent years- single nuclei RNA sequencing (snRNA-seq)^16^, to study specific transcriptome alterations in the hippocampus after bTBI in greater depth, so as to provide a powerful reference map. This approach allows for the independent RNA expression profiling of each nucleus by sequencing the RNA at the individual cell level. As a result, we can gain a deeper understanding of the state, gene expression changes, and interaction mechanisms of different types/ sub-type of cells in the hippocampus after bTBI^17^. Based on a well-established animal model of bTBI, snRNA-seq was performed on murine hippocampus, and a high-quality dataset of bTBI was obtained for the first time. Here, we demonstrated that the dataset has the sequencing depth required for in-depth analysis, and used strict standards to control its quality. Additionally, the dataset’s high quality and reliability were confirmed through cell clustering, cell type annotation, differentially expressed gene analysis, Kyoto Encyclopedia of Genes and Genomes (KEGG) signal pathway enrichment analysis, pseudotime analysis, combined with previous literature reports. This dataset can be used to identify the cell types/sub-types of hippocampus, discover new marker genes, examine transcriptomic changes in distinct cell types, and characterize the differentiation routes of NSCs after bTBI. Therefore, this data is suitable for those interested in exploring hippocampal cell types, the mechanisms and pathogenesis of secondary injury after bTBI, as well as related NSC differentiation, with significant reference value for mining specific targets for the diagnosis or treatment of bTBI.

## Methods

### Animals

All animal experimental procedures were carried out in accordance with local laws and institutional guidelines, with approval granted by the Experimental Animal Ethics Committee of West China Hospital, Sichuan University (Approval Number: 2020386 A). Furthermore, these procedures were executed following the principles delineated in the guide for the Care and Use of Laboratory Animals of the National Institutes of Health (NIH publication #85-23, revised in 1985). C57BL/6 J mice aged between 7–10 weeks (n = 3/group) were selected for the study. Mice were kept under standard conditions of 12-hour light-dark cycle, temperature 22–25 °C, and relative humidity 40–60%, with ad libitum access to food and water. The experimental animals were sourced from ENSIWEIER Bio-Technology Co., Ltd. in Chengdu.

### Blast-traumatic brain injury

In this experiment, C57BL/6 J mice aged 7–10 weeks were randomly assigned to either a bTBI or a sham-operated group, with 3 mice in each group. They were housed in a room with a 12-hour light-dark cycle and controlled temperature. Prior to surgery, the mice were anesthetized with intraperitoneal injection of pentobarbital (5 mg/100 g ip) and immobilized to prevent movement. In the bTBI group, a BST-I biological shock tube driven by compressed gas was used to expose the mice to a 5.0 MPa blast shock wave, created by rupturing an aluminum sheet with high-pressure compressed gas, to simulate open-field conditions and construct the bTBI injury model^18^. In contrast, the sham-operated group was anesthetized and placed near the blast chamber, but was not exposed to the blast shock wave. After the blast shock, all mice were returned to a 12-hour light-dark cycle, temperature-controlled room for housing after they regained consciousness. After 48 hours of injury, 4% isoflurane was used for anesthesia, followed by 1–2% isoflurane for maintenance anesthesia. The mouse brain tissue from the hippocampal region was micro-dissected (n = 3/group) after cardiac perfusion of 20–25 ml of PSB treated with hypothermia until the mouse liver turned pale. The brain tissues were washed in pre-chilled PBS to remove any residual blood and placed in liquid nitrogen for rapid freezing before being transferred to −80 °C for subsequent experiments.

### Neurobehavioral assessment

At 6 hours after bTBI, we conducted the Modified Neurological Severity Score (mNSS) test on the mice to evaluate the degree of neurological impairment and behavioral functional deficits. This test involved motor, sensory, reflex, and balance assessments, with scores ranging from 0 to 18. A higher score indicates more severe neurological impairment, while a score of 0 indicates normal neurological function with no deficits. A maximum score of 18 indicates loss of consciousness or death^19^.

### Nuclei isolation

The frozen hippocampus was treated with NLB buffer (0.2 U/μL RNase Inhibitor (Takara, Kyoto, Japan), 250 mM sucrose, 10 mM Tris-HCl, 3 mM MgAc2, 0.1% Triton X-100 (Sigma-Aldrich, St Louis, MO, USA), 0.1 mM EDTA) for purification of nuclei using density gradient centrifugation with sucrose solutions of varying concentrations. The resulting nuclei were visually inspected for appearance and cell lysis using trypan blue, and the concentration was adjusted to 1,000 nuclei/μL for snRNA-seq.

### Single nucleus RNA sequencing

cDNA synthesis, library construction, and single-nucleus RNA sequencing were carried out by NovelBio Co., Ltd. located in Shanghai, China. The experiment utilized the Chromium Single Cell 3′ Reagents Kits v3.1 (10x Genomics, Pleasanton, USA). Sequencing was performed on a 10x Genomics Chrome Controller instrument in strict accordance with the Chrome Single Cell 3′ Reagent Kit v3.1 user guide. Briefly, after washing twice with 1 × PBS + 0.04% BSA, nuclei at a concentration of 1,000 nuclei/μL were added to the 10 × Genomics Chromium Controller machine to generate Gel Beads. A controller machine was used to produce Gel Beads-in-Emulsion (GEM) for the experiment. The mRNA was prepared using the 10x Genomics Chromium Single Cell 3′ reagent kit featuring V3.1 chemistry. During this step, nuclei were combined with oligonucleotide-coated Gel Beads in the GEM. The oligonucleotides included poly-dT sequences and specific barcodes for each cell and transcript, which enabled the mRNA to be released after nucleolysis within the droplet. after reverse transcription, the barcoded-cDNA is released from the GME and purified. Once the library preparation was deemed sufficient, the Qubit High Sensitivity DNA assay (Thermo Fisher Scientific, Waltham, MA, USA) was applied to quantify the final libraries. Additionally, the quality and concentration of the final library were evaluated using Bioanalyzer 2200 (Agilent Technologies, Santa Clara, CA, USA). Finally, all libraries were subjected to a 150 bp paired-end run using the Novaseq. 6000 (Illumina, San Diego, CA, USA).

The raw data was converted into FASTQ files and analyzed using CellRanger v3.1.0. We compared the short reads with the mouse reference genome (GRCm38 Ensembl: version 92) and generated feature-barcode matrices containing the barcode and unique molecular identifier (UMI) counts.

### Normalization, clustering and data visualization

We used the Seurat R package to conduct the following analysis^20^. First, we normalized the UMI counts for each gene via dividing the UMI counts by the total number of UMIs for the cell, multiplying by 10,000, and then log-transforming. We subsequently subjected the nuclei to a cell-level quality control procedure. For the percentage of mitochondrial transcripts detected in each cell (percent.mt), the number of genes detected in each nucleus (nFeature_RNA), and the total number of detected mRNA molecules detected in the nucleus (nCount_RNA), we established stringent thresholds (nFeature_RNA per cell must be higher than 200 and be less than 10,000; percent.mt must be less than 10%; nCount_RNA be less than 100,000.). Next, we identified the 2,000 highly variable genes using the FindVariableFeatures function in the Seurat R package. We performed principal component analysis (PCA) to reduce the dimensionality of the dataset using the RunPCA function in the Seurat R package. We plotted the cumulative standard deviation of each PC using the PCElbowPlot function in the Seurat R package to identify the inflection point, which indicated the number of PCs that explained the majority of the variance. We used the ScoreJackStraw function in the Seurat R package to identify the significance of each gene in association with each PC and obtained the optimal number of principal components (PCs) for cluster analysis and visualization. We used the FindNeighbors function with the top10 PCs and the FindClusters function with a resolution of 0.8 in the Seurat R package for unsupervised clustering of the dataset. The other parameters were set as the default parameters. The clusters were then visualized using the Uniform Manifold Approximation and Projection for Dimension Reduction (UMAP) method. Finally, marker genes were automatically calculated by Wilcoxon rank sum test using the FindAllMarkers function in the Seurat R package.

### Pseudotime analysis

Reprogramming trajectory analysis of neural stem cells (NSCs) was conducted using Monocle2. This visualizes the pseudotime trajectory as a tree structure in reduced dimensional space. In this paper, we used the differentialGeneTest function (with a q-value threshold of < 0.05) in the Monocle2 R package to identify significantly changed genes. Heatmaps were used to show gene sequences with certain expression patterns along the trajectory direction. Based on Pseudotime analysis, we analyzed and showed some of the fate-specific genes that affect the fate of cell developmental processes at branching points on single-cell trajectories using the branching expression analysis model (BEAM).

### Functional annotation and pathway analysis

We conducted Gene Ontology (GO) and Kyoto Encyclopedia of Genes and Genomes (KEGG) pathway analyses using the clusterProfiler R package^21^. Fisher’s exact test was employed to determine the GO categories and pathways, and the p-values were corrected using the false discovery rate (FDR). Enrichment items with a p-value < 0.05 were considered to be significantly enriched.

## Data Records

The sequencing raw data has been uploaded to Gene Expression Omnibus (GEO) (project number: GSE230253^22^; GSM7210822 for Sham; GSM7210821 for bTBI). We also uploaded the data to figshare as matrix.mtx.gz, features.tsv.gz and barcodes.tsv.gz in a matrix format, respectively (10.6084/m9.figshare.22659415 for Sham^23^; 10.6084/m9.figshare.22659406 for bTBI^24^). Through the raw data we provide, the FASTQ files can be processed using Cell Ranger to generate BAM files.

## Technical Validation

**Fig. 1:**
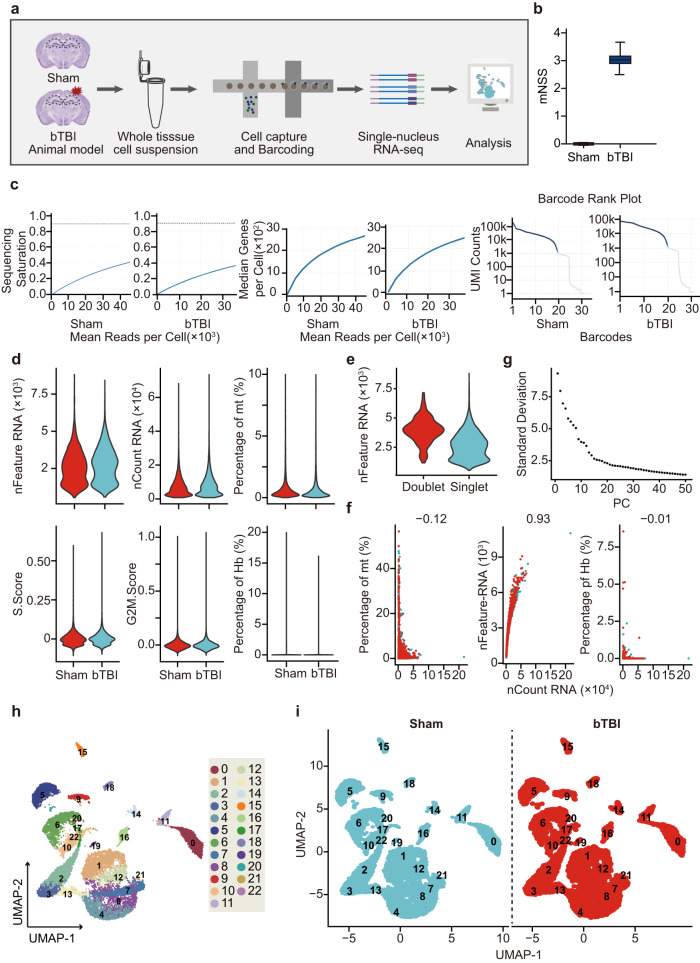
Workflow and quality control. (**a**) Summary of experimental design. (**b**) Neurobehavioral assessment by mNSS test results. (**c**) Raw data of the Single nucleus RNA sequencing. Left: Sequencing Saturation, Mid: Median gene per cell, Right: Barcode Rank Plot. (**d**) Single nucleus RNA sequencing quality control. (**e**) The nFeature RNA for single or double cell. (**f**) The correlation coefficient between Percentage of mt(left), Feature RNA(mid),Percentage pf and nCount RNA. (**g**) The correlation coefficient between Standard Deviation and PC values. (**h**) UMAP plots of the hippocampal cells. (**i**) UMAP plots of Sham and bTBI.

In this investigation, we utilized the single-nucleus RNA sequencing (snRNA-seq) technique to sequence individual hippocampal nuclei from both the bTBI and sham groups of mice (Fig. 1a). The analysis of modified Neurological Severity Score (mNSS) at 6 hours post bTBI revealed a significant disparity between the bTBI group and the sham group, indicating the impact of bTBI (Fig. 1b). The 10x Genomics Chromium platform was used to construct a snRNA-seq library from the hippocampus of bTBI mice and the sham control. Subsequently, sequencing and bioinformatic analysis were performed. In the bTBI and sham operation groups, 9823 and 7834 nuclei were involved, respectively. Per group, the median UMI counts detected in each nucleus were 6568 and 5801, and the average gene readings in each nucleus were 45667 and 36326, and the average gene counts were 2658 and 2480, respectively. The saturation curves for the 2 groups shows that there is a similar distribution and very small technical differences in data quality metrics between the bTBI and sham operation groups (Fig. 1c). These sequence saturation curves are based on the notion of sequencing saturation. The detailed calculation method for sequencing saturation can be accessed on the 10x Genomics official website (https://kb.10xgenomics.com/hc/en-us/articles/115003646912).

To eliminate the potential low-quality results that may be caused by cell disruption, apoptosis, or other technical factors, dead cells need to be removed from subsequent analysis. After cell death, RNA leaks out of the cytoplasm due to increased cell permeability and membranolysis, while mitochondrial transcripts are retained, resulting in an increase in the percentage of mitochondrial transcripts. Therefore, cells with a high percentage of mitochondrial transcripts (percent.mt) were interpreted as dead cells. Additionally, nuclei from dead cells often have a much lower number of genes detected than normal cells. Therefore, we set strict thresholds for percent.mt and the number of genes (nFeature_RNA) detected in each nucleus (Fig. 1d), to ensure high data quality. Specifically, we required that nFeature_RNA per nucleus be higher than 200 and percent.mt be less than 10%. We also required that nFeature_RNA be less than 10,000 and nCount_RNA be less than 100,000, as excessive gene numbers are often indicative of double nuclei. Furthermore, we used the DoubletFinder R package to screen for double nuclei and remove them (Fig. 1e). Following these screening steps, we retained 17,278 high-quality nuclei, with 9,639 and 7,639 nuclei in the bTBI and sham operation groups, respectively. The correlation coefficient between percent.mt and nCount_RNA was −0.12, and the correlation coefficient between percent.hb and nCount_RNA was −0.01 in the bTBI and the sham control, indicating that mitochondrial transcripts and erythrocyte genes did not change with increasing sequencing depth. At the same time, the correlation coefficient between nFeature_RNA and nCount_RNA was 0.93, suggesting that the number of genes detected increased with deepening sequencing depth (Fig. 1f). These above results further demonstrate that our data correspond to living cells. In conclusion, by performing the above screening steps, we obtained high-quality single-nucleus transcriptomic datasets from the hippocampus of bTBI mice and sham control.

**Fig. 2:**
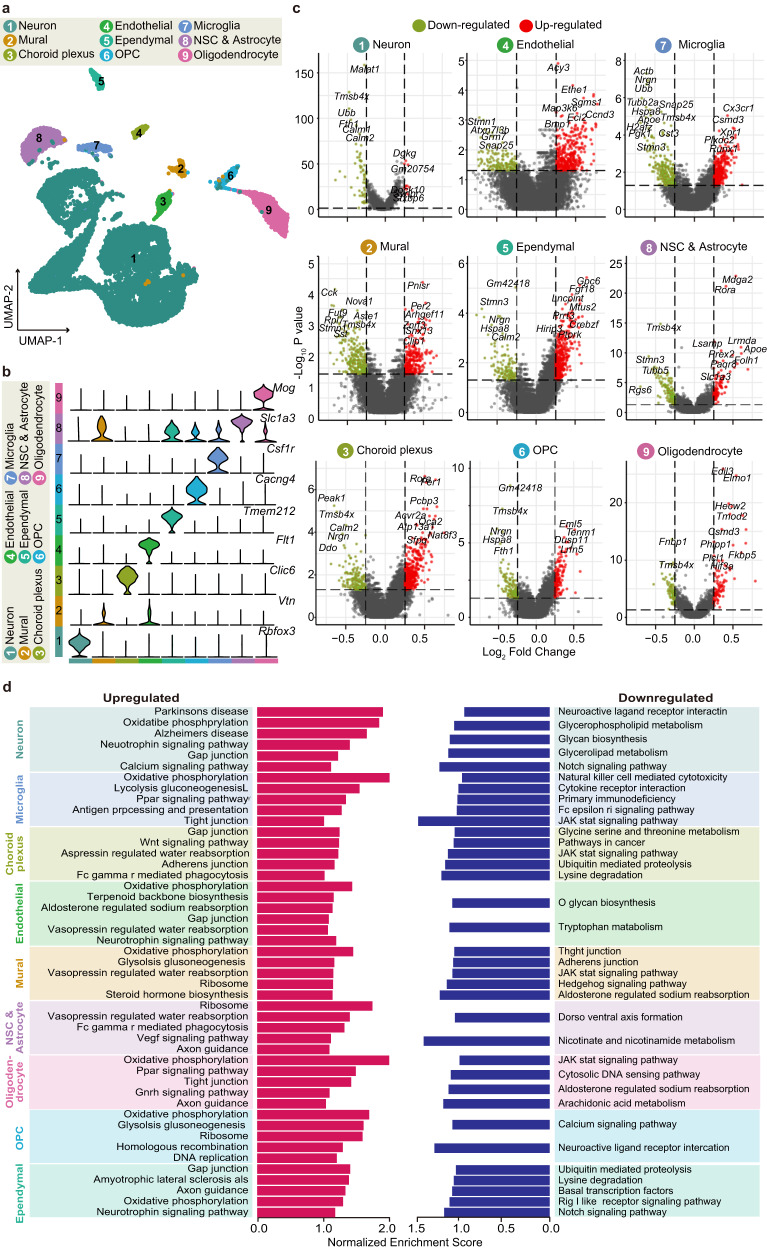
Difference analysis between the Sham and the bTBI hippocampus. (**a**) UMAP plot showing the annotation of hippocampal cells. (**b**) The expression of marker genes in each type of cells. (**c**) Gene expression in bTBI group compared with Sham. (**d**) The Kyoto Encyclopedia of Genes and Genomes (KEGG) enrichment analysis results.

To further analyze the cell population and assess the utility value of our dataset, we employed the principal component analysis (PCA) (Fig. 1g). Using unsupervised calculation, the FindClusters function in the Seurat R package divided the nuclei into 23 clusters, and UMAP projected all sequenced nuclei into a 2-dimensional space (Fig. 1h). Our results showed that the data from bTBI and sham operation groups had similar distribution on the 2-dimensional map, indicating that we successfully removed the influence of batch effects after our previous quality control steps (Fig. 1i). Based on known specific markers, the hippocampal nuclei were classified into 9 major cell types: neurons, mural cells, choroid plexus cells, endothelial cells, ependymal cells, oligodendrocyte precursor cells (OPCs), microglia, NSC & astrocytes, and oligodendrocytes^25–30^ (Fig. 2a). The marker gene *Rbfox3*, which is specifically expressed by neurons, was used to annotate fifteen clusters as neurons. Additionally, *Syt1*, *Meg3*, and *Snap25*, which are highly expressed in neurons, were also found to be highly enriched in these clusters, further supporting our annotation results. The marker gene *Vtn*, which is commonly found in mural and endothelial cells, was highly enriched in cluster 14 and 18. Notably, mural marker genes such as *Acta2* and *Tagln* were highly expressed in cluster 14, while the classical endothelial marker gene *Flt1* was highly enriched in cluster 18, leading us to annotate cluster 14 as mural cells and cluster 18 as endothelial cells. Cluster 16 was annotated as choroid plexus cells by a specific marker gene *Clic6*. The presence of the *Tmem212* in cluster 15 indicated that this cluster was ependymal cells. Cluster 11 was highly enriched with *Pdgfra*, which is often considered a specific marker gene for OPCs in previous literature, so it was annotated as OPCs. The presence of the specific marker gene *Csf1r* in cluster 9 led us to define it as microglia cells. Cluster 0 was highly enriched with the oligodendrocyte cell-specific marker gene *Mog*. At the same time, we also noticed that the genes *Mbp*, *Opalin*, and *Plp1*, which were previously used to annotate oligodendrocyte cells, were also highly enriched in this cluster, so we annotated cluster 0 as oligodendrocyte cells. There were many similar molecular characteristics NSCs and astrocytes. At present, a large number of studies held the viewpoint that hippocampal NSCs and astrocytes share some molecular characteristics and astrocytes exhibit stem cell properties under certain injury conditions, so we preliminarily grouped them into a large category^31,32^. We found that the marker gene *Slc1a3* was highly enriched in cluster 5, and the marker genes *Aqp4* and *Egfr*, which were previously used to annotate NSCs and astrocytes, were also enriched in the cluster, so cluster 5 was annotated as NSC & Astrocytes (Fig. 2b).

To further validate the data have certain value in studying the transcriptomic changes after bTBI, we used the FindMarkers function and Wilcox rank sum test to screen for the 10,568 differentially expressed genes (DEGs) across the 9 cell types, using predefined criteria (min.pct = 0.01, logfc.threshold = 0.01). We defined DEGs with p_val < 0.05 and |log2FC| > 0.25 as significant. Among them, significantly up-regulated genes were defined as those with log2FC > 0.25, while significantly down-regulated genes were defined as those with log2FC < −0.25. We utilized these criteria to identify significant DEGs across the 9 cell types, presenting the subset of genes for each type, and performed KEGG analyses on the DEGs within each cell type (Fig. 2c,d). The functional analysis of those DEGs further supports the high quality of our dataset. For instance, after bTBI, the expression of *Apoe* was up-regulated in NSC & Astrocyte, which is consistent with the reported mechanism of *Apoe* mediating hippocampal neurogenesis induced by TBI^33^. Moreover, blood-brain barrier (BBB) injury is a common feature of secondary brain injury after TBI. In our dataset, we observed that *Tjp1* (Z0-1), a gene related to tight junction protein (TJP) expression and essential for BBB function, was significantly down-regulated, which is consistent with the findings reported in previous studies, thus further supporting the reliability of our dataset^34^. Additionally, microglia, as the cellular component of the innate immune system in brain, showed significant up-regulation of *Cx3cr1* after injury, suggesting an activation of the inflammatory response, which aligns with the reported mechanism of microglia participating in neuroinflammatory responses after TBI^35–37^. Overall, our results demonstrate the reliability of our data and analysis, providing references for future bTBI researches.

**Fig. 3:**
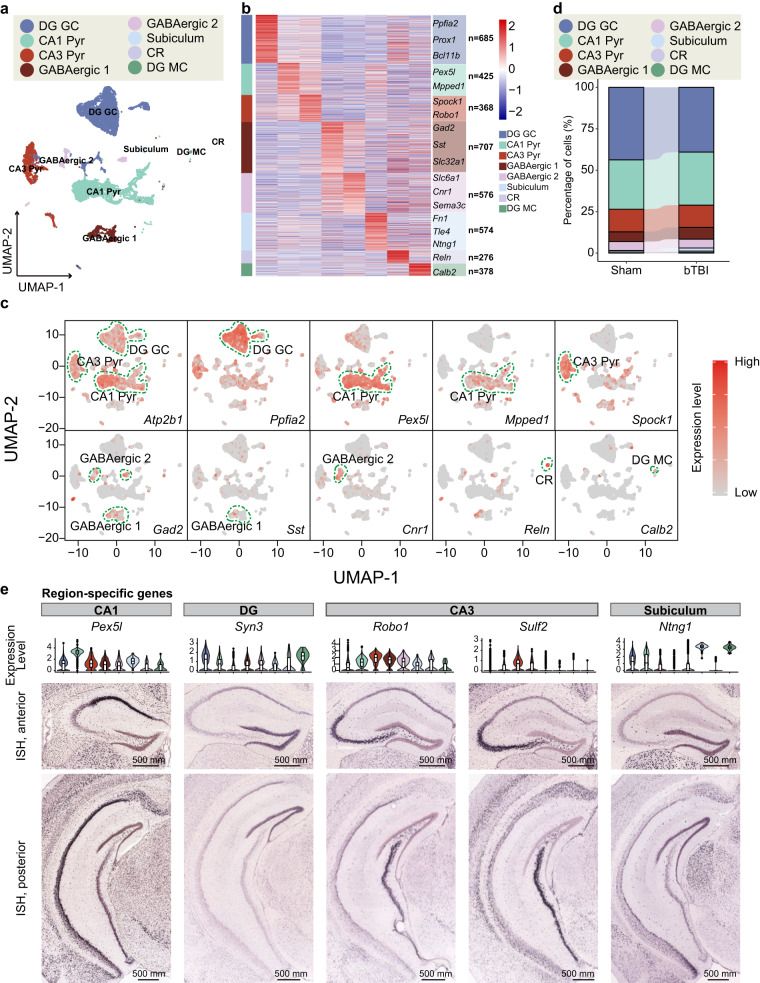
Analysis of subclasses of hippocampal neuronal cells. (**a**) UMAP plot of hippocampal neuron in the hippocampal niche (DG GC, CA1 Pyr, CA3 Pyr, GABAergic 1, GABAergic 2, Subiculum, CR and DG MC). (**b**) Heatmap showing differentially expressed genes catalogued in eight cell types. (**c**) Distribution of marker genes for annotation: *Atp2b1*, *Ppfia2* (DG GC); *Pex5l*, *Mpped1*, *Atp2b1* (CA1 Pyr); *Gad2*, *Sst* (GABAergic 1); *Gad2*, *Cnr1* (GABAergic 2); *Reln* (CR); *Calb2* (DG MC). (**d**) The proportion of nine subtypes of cells in Sham group and bTBI group. (**e**) Region-specific genes.

Subsequently, we conducted a more detailed analysis focusing on neurons. We used the FindAllMarkers function in the Seurat R package (min.pct = 0.25, logfc.threshold = 0.25) and identified 3,989 marker genes specific to neuronal cells. Based on published literature on known markers, the neuronal clusters fell into 8 sub-types: granule cells in dentate gyrus (DG GC), pyramidal neurons in cornu ammonis 1 (CA1 Pyr), pyramidal neurons in cornu ammonis 3 (CA3 Pyr), two types of GABAergic neurons (GABAergic 1 and GABAergic 2), Subiculum, Cajal-Retzius cell (CR), and DG mossy cell (DG MC)^38–43^ (Fig. 3a). Marker genes of different neuronal sub-types were displayed using the heatmap (Fig. 3b). To further validate the reliability of our annotation, we visualized the distribution of the marker genes for each sub-type on UMAP (Fig. 3c). Our analysis revealed specific distribution of *Ppfia2* in DG GC cells, enrichment of *Pex5l* and *Mpped1* in CA1 Pyr cells, specific expression of *Spock1* in CA3 Pyr cells, and high expression of *Fn1*, *Reln*, and *Calb2* in Subiculum cells, CR, and DG MC cells, respectively, which support the reliability of our data and annotation results. Furthermore, we found that *Atp2b1* was a specific marker for glutamine excitatory neurons, as it was widely distributed in DG GC cells, CA1 Pyr cells, and CA3 Pyr cells. The specific marker gene for inhibitory GABAergic neurons- Gad2, was widely distributed in GABAergic 1 cells and GABAergic 2 cells, while with higher expression in GABAergic 2 cells. In addition, the specific marker genes *Sst* and *Cnr1* were highly expressed in GABAergic 1 cells and GABAergic 2 cells, respectively, further supporting our annotation results. Notably, we observed a significant reduction in the percentage of DG GC cells among all the hippocampal neuronal sub-types after bTBI. This finding is consistent with previous reports of secondary damage to hippocampal DG neurons after bTBI^44,45^ (Fig. 3d). Additionally, we also identified novel markers for various sub-types of hippocampal neurons, such as *Pex5l* for CA1, *Syn3* for DG, *Robo*1 and *Sulf2* for CA3, and *Ntng1* for Subiculum. To verify the reliability of these novel marker genes, we examined their locations in the hippocampus using the Allen Brain Map database^46^ (Fig. 3e). We found that the expression of these markers locationally matched the corresponding neuronal sub-types, providing further support for the quality and reliability of our data and analysis.

**Fig. 4:**
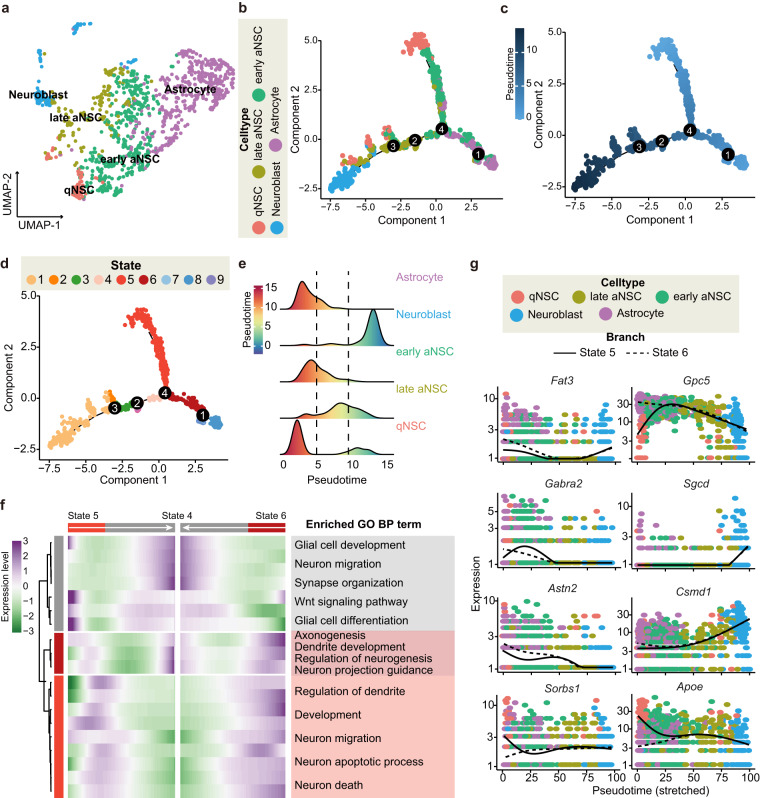
Pseudotime analysis of NSCs in the hippocampus following bTBI. (**a**) UMAP plot showing the annotation of NSCs subtype cells. (**b**) The panel showing cells from different NSCs sub types along the pseudotime trajectory. (**c**) Pseudotime trajectory results of NSCs with DDRTree method for dimension reduction. (**d**) Cell ordering from different differentiation stages along the pseudotime trajectory. (**e**) The main cell types corresponding to different time periods. (**f**) Left: Fate-specific gene expression Heatmap at branching point 4. Right: Biological processes for each cluster. (**g**) Expression pattern of *Fat3*, *Gpc5*, *Gabra2*, *Sgcd*, *Astn2*, *Csmd1*, *Sorbs1*, *Apoe* in the reprogramming route of the NSC&Astrocyte. Branch Y_10: state 5; Branch Y_45: state 6.

To visualize the reprogramming route of the NSC & Astrocyte in the hippocampus following bTBI, we grouped and annotated the detailed types of those nuclei at the first step. Using the Seurat R package’s FindCluster, the “NSC & Astrocyte” were divided into 11 clusters unsupervisedly. Based on known markers, we grouped those clusters into 5 classes- astrocytes, quiescent NSCs (qNSCs), early active NSCs (aNSCs), late aNSCs, and neuroblasts, and projected them into a 2-dimensional space using the UMAP^28,29,47,48^ (Fig. 4a). Pseudotime trajectories (Fig. 4c) were established for all NSC & Astrocyte using the Monocle2 R package. We identified 4 different branches and 9 different states on this route. At the 4 branch points, cells underwent specific gene expression patterns that determined their cell fate during differentiation (Fig. 4d). We observed that at branch point 4, state 5 (mainly composed of qNSCs and early aNSCs) and state 6 (mainly composed of astrocytes and early aNSC cells) converged to develop into state 4 (mainly composed of early aNSCs), which was then differentiated into state 3 (mainly composed of late aNSCs), and finally into state 1 (mainly composed of late aNSCs and neuroblasts) (Fig. 4b–e). This is consistent with the processes of post-injury neurogenesis reported in previous literature^49–51^. Collectively, these results further demonstrate the reliability of our dataset.

To display the detailed information on the gene expression per branch, we selected the characteristic genes in each type of NSC & Astrocyte (gene expression greater than 0.1, expressed in at least 10 cells). Based on this, the NSC & Astrocyte was clustered and dimensionally reduced. Subsequently, differentialGeneTest function was introduced to obtain 1,265 fate-specific genes under different states via unsupervised clustering. Additionally, we presented genes at branch point 4 (Fig. 4f) which may have critical roles in the cell fate determination in the differentiation from the qNSC cells (state 5) and astrocytes (state 6) into early aNSCs (state 4). The heatmap of fate-specific genes determining this process was divided into 3 clusters, and GO enrichment analysis was conducted on the genes within each cluster. Results showed that these genes mainly aggregate in glial cell development, glial cell differentiation, dendrite development, and neuron projection guidance, which is consistent with published literature on the mechanism of neurogenesis after TBI^52^. Finally, we ranked fate-specific genes by p-value and q-value and displayed the top 8 fate-specific genes (Fig. 4g) at branch point 4. Overall, the above analysis indicates that our dataset is of high quality and utility, providing valuable insights into the mechanisms of hippocampal neurogenesis following bTBI and potential targets for promoting neurogenesis.

## Usage Notes

Our raw data bits, based on UMI’s 10x Genomics sample, were uploaded to the database in.fastq format. Various tools, including cellranger (v3.1.0), UMI-tools (v1.0.0), and zUMIs (v2.4.5), can be used to process the raw data. Read10X function in the Seurat R package can be used to generate gene-barcode matrices.

The R packages mentioned in this manuscript, such as Seurat, Monocle2, clusterProfiler, org.Mm.eg.db and others, can be used to perform basic analyses such as quality control, differential expression analysis, pseudotime analysis and GO analysis on the gene-barcode matrices. Matrices could also be processed using other common analysis methods such as Single Cell Regulatory Network Inference and Clustering (SCENIC) and CellChat, which were not discussed in this manuscript. The corresponding code scripts of the analysis mentioned in this study can be accessed in the code availability.

Based on the above-mentioned analysis methods or other analysis methods, this dataset can be used to: (1) explore the heterogeneity of mouse hippocampal cells and identify novel cell types/ sub-types; (2) discover new specific markers for different hippocampal cell types; (3) study the gene expression of hippocampal cells to provide a reference map for subsequent functional researches; (4) investigate the specific gene expression alterations of various types of hippocampal cells after bTBI; (5) reveal the changes in crosstalk between different cell types of mouse hippocampus following bTBI; (6) analyze the dynamic changes of the NSC reprogramming process after bTBI, and its regulatory mechanisms; (7) provide a reference map for the study of the secondary injury and repair mechanisms of bTBI, and identify the diagnostic and therapeutic targets of bTBI.

It is worth noting that snRNA-seq has certain limitations. Compared to single-cell RNA sequencing, snRNA-seq loses transcriptional information in the cytoplasm. At the same time, compared to bulk RNA sequencing, snRNA-seq has a larger and more complex background noise. This issue may require the development of new algorithms to ensure data reproducibility.

## Data Availability

All of the programs we use in this study for analysis are based on open data sets. The version information of the open program and its official website: 1. FastQC (v0.11.9) https://github.com/s-andrews/FastQC. 2. CellRanger (v3.1,0) https://github.com/10XGenomics/cellranger. 3. Seurat (v3.1.4) https://satijalab.org/seurat. 4. ClusterProfiler (v4.6.2) https://bioconductor.org/packages/release/bioc/html/clusterProfiler.html. 5. Monocle2 (v2.4.0) http://cole-trapnell-lab.github.io/monocle-release.
